# The BISAP score, NLR, CRP, or BUN: Which marker best predicts the outcome of acute pancreatitis?

**DOI:** 10.1097/MD.0000000000028121

**Published:** 2021-12-23

**Authors:** Greta Maria Dancu, Alina Popescu, Roxana Sirli, Mirela Danila, Felix Bende, Cristi Tarta, Ioan Sporea

**Affiliations:** a Department of Internal Medicine II, Division of Gastroenterology and Hepatology, Center for Advanced Research in Gastroenterology and Hepatology “Victor Babes” University of Medicine and Pharmacy Timisoara, Romania; b Department X, 2nd Surgical Clinic, Researching Future Chirurgie 2, Victor Babes University of Medicine and Pharmacy, Timisoara, Romania.

**Keywords:** Biomarkers, pancreatitis, prediction, severity

## Abstract

Acute pancreatitis is a common disease, and the mortality rate can be high. Thus, a risk assessment should be performed early to optimize treatment. We compared simple prognostic markers with the bedside index for severity in acute pancreatitis (BISAP) scoring system to identify the best predictors of severity and mortality.

This retrospective study stratified disease severity based on the revised Atlanta criteria. The accuracies of the markers for predicting severe AP (SAP) were assessed using receiver operating characteristic curves. The sensitivity, specificity, positive predictive value, and negative predictive value were calculated for each marker. Multivariate logistic regression analyses were used to identify independent predictors of SAP and mortality.

The area under the curve (AUC) for the BISAP score was classified as fair for predicting SAP. The neutrophil-to-lymphocyte ratio at 48 hours (NLR48 h) and the C-reactive protein level at 48 hours (CRP48 h) had the best AUCs and were independently associated with SAP. When both criteria were met, the AUC was 0.89, sensitivity was 68%, and specificity was 92%. CRP48 h and hematocrit at 48 hours were independently associated with mortality.

NLR48 h and CRP48 h were independently associated with SAP but not superior to the BISAP score at admission. Assessing NLR48 h and CRP48H together was most suitable for predicting SAP. The CRP level was a good predictive marker for mortality.

## 1 Introduction

Acute pancreatitis (AP) is one of the most common gastrointestinal tract diseases characterized by a rapid inflammation of the pancreas,^[1]^ potentially involving the surrounding tissues and distant organs or systems. Annually, 270,000 patients are hospitalized due to AP in the United States, and the in-hospital treatment costs are higher than US$ 2.5 billion per year.^[2]^

The overall mortality rate for mild forms of AP is 5% and 1.5%. However, the rate increases to between 17% and 30%^[3]^ for severe forms, with some reports of up to 50%.^[4]^ As such, a risk assessment should be performed to stratify patients into high- or low-risk categories to assist with triage. Further, patients with organ dysfunction should be admitted to an intensive care unit or intermediary care setting when the signs indicate severe disease.^[5]^ A series of severity scoring systems have been proposed and accepted for the early identification of patients with severe disease. Among them, the Acute Physiology and Chronic Health Evaluation II system,^[6]^ Ranson criteria,^[7]^ and the bedside index for severity in acute pancreatitis (BISAP)^[8]^ score are the most widely used in routine clinical practice. However, none are sensitive or specific enough, and there has been no definitive consensus as to which scoring system should be used. Currently, most classical methods for assessing AP severity have limitations. Namely, most are not simple, rapid, or economical.^[9]^

Changes in peripheral blood components are used to determine the prognosis of many diseases.^[10]^ For example, hematocrit (Ht), the red cell distribution width (RDW),^[11]^ the neutrophil-to-lymphocyte ratio (NLR), and the platelet-to-lymphocyte ratio (PLR) have been reported to correlate with AP severity.^[12]^ Further, blood urea nitrogen (BUN) could be a severity indicator for any metabolic disarray leading to alterations in urea production (accelerated tissue catabolism), absorption (gastrointestinal bleeding), or excretion (poor renal perfusion).^[13,14]^ Of the inflammation markers, C-reactive protein (CRP) is the most used, and many studies have identified a correlation between high CRP levels and severe AP.^[15]^

This study analyzed the predictive value of these simple, single parameters for developing severe AP (SAP) compared with BISAP, the relatively mature scoring system. Further, we examined the predictive value of these parameters for the overall complication rate and mortality in patients with AP.

## 2 Methods

### 2.1 Patients

This retrospective study included all patients admitted to a tertiary department of gastroenterology with AP between January 1, 2018 and June 30, 2019. The patients were retrospectively identified using the 10th revision of the International Statistical Classification of Diseases and Related Health Problems (i.e., ICD-10) code for acute pancreatitis.

AP was diagnosed if 2 of the following criteria were met: consistent abdominal pain, a serum lipase level 3-fold higher than the normal level, and typical aspects of AP were observed on computed tomography images.

Patients with chronic pancreatitis (indicated by intraductal calculi, ductal stricture, or parenchymal calcification) were excluded. Patients with infection at presentation (e.g., cholangitis, cholecystitis, or pneumonia), pregnant women, and patients younger than 18 years were also excluded.

### 2.2 Disease classification

Patients were stratified by disease severity using the revised Atlanta Criteria^[16]^:

1. Mild AP (MAP): no organ failure and no local or systemic complications.
2. Moderately severe AP (MSAP): transient organ failure (resolved within 48 hours) or local complications.
3. SAP: persistent organ failure (longer than 48 hours).

Local complications included acute peripancreatic fluid collection, pancreatic pseudocyst, acute necrotic collection, and walled-off necrosis. Organ failure was defined as a score of 2 or more using the modified Marshall scoring system for the renal, cardiovascular, or respiratory organ system.

### 2.3 Data collection

The following were collected from the patients’ charts: sex, age, blood pressure (mm Hg), respiratory rate (breaths per minute), oxygen saturation (%), pulse rate (beats per minute), the BISAP score at admission, the creatinine level at admission and 48 hours (mg/dL), Ht at admission and 48 hours (%), the CRP level at 48 hours (mg/dL), NLR at admission and 48 hours (NLR48 h), RDW at admission (%), BUN at admission and 48 hours (mg/dL), PLR at admission and 48 hours (PLR48 h), and the glucose level at admission (mg/dL). The etiology, morbidity, and mortality data were also collected.

Laboratory data were obtained by spectrophotometry. NLR and PLR were calculated at admission and 48 hours using the international criteria (NLR = neutrophil count/lymphocyte count, PLR = platelet count/lymphocyte count).

The BISAP score was evaluated at admission using the worst parameters available in the first 24 hours. The 5-point BISAP score system incorporates the BUN level (>25 mg/dL), impaired mental status, systemic inflammatory response syndrome, age >60 years, and the presence of pleural effusion. One point was assigned per variable within 24 hours of presentation and added to make a composite score of 0 to 5.^[17]^ Patients were followed up for 90 days after discharge through outpatient service visits.

### 2.4 Statistical analyses

Variables are expressed as medians (ranges) and categorical data as percentages, as appropriate. The accuracy of each marker to predict SAP and mortality was assessed using receiver operating characteristic (ROC) curves using MedCalc 15.0 (MedCalc Software, Ostend, Belgium). The sensitivity (SE), specificity (SP), positive predictive value (PPV), and negative predictive value (NPV) were also calculated. A 2-tailed *P* value < .05 was considered statistically significant. We selected the best predictors for SAP based on the ROC results, then used multivariate logistic regression analyses to assess whether the inflammation markers were independent factors for predicting SAP in AP patients. Positive likelihood (LR+) and negative likelihood (LR−) ratios were calculated as follows: LR+ = SE/100-SP; and LR− = 100-SE/SP.

To assess the effect of parameter changes over time, we calculated the Δ(parameter), equaling the parameter value at admission minus the parameter value at 48 hours. The usefulness of Δ(parameter) for predicting SAP was analyzed using the ROC curve.

### 2.5 Ethics approval

Our study was approved by the Local Committee of Ethics for Scientific Research of the Regional Emergency Hospital Timisoara.

## 3 Results

**Figure 1:**
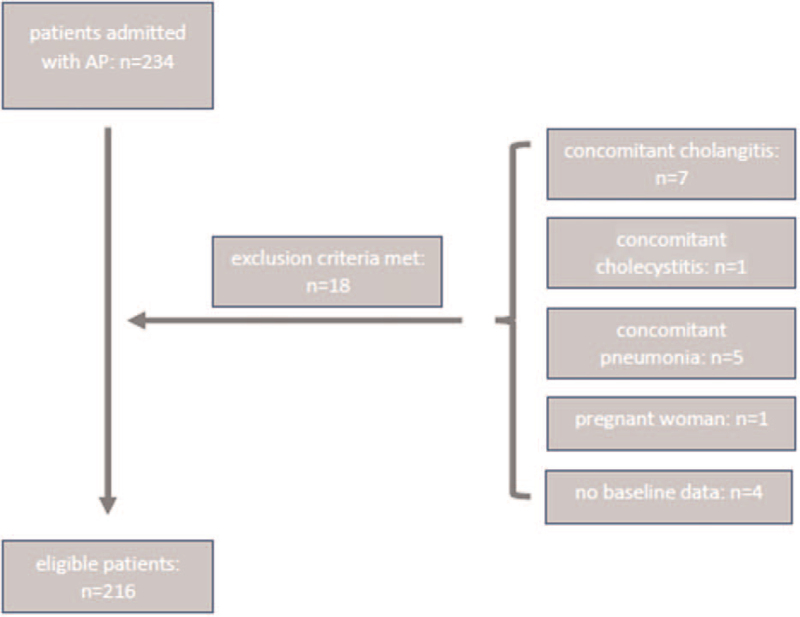
The patient selection flowchart outlines the number of patients admitted with acute pancreatitis, the number of patients excluded, and the final number included in the study. AP = acute pancreatitis.

During the study period, 234 patients were admitted for AP; 18 were excluded owing to unavailable baseline data or meeting the exclusion criteria. In total, 216 patients were enrolled (Fig. 1).

**Figure 2:**
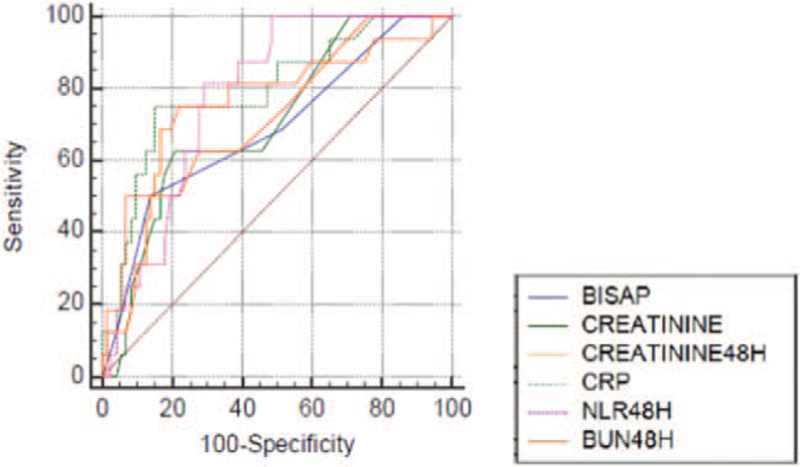
A graphical representation of the AUCs of the BISAP scale, the creatinine levels at admission and 48 hours, CRP48 h, NLR48 h, and BUN48 h for predicting SAP. AUC = area under the curve, BISAP = bedside index of severity in acute pancreatitis, BUN48 h = the blood urea nitrogen level at 48 hours, CRP48 h = the C-reactive protein level at 48 hours, NLR48 h = the neutrophil-to-lymphocyte ratio at 48 hours, SAP = severe acute pancreatitis.

Table 1 summarizes the demographic data, etiology, mortality rate, and in-hospital outcomes based on severity, and Table 2 presents the laboratory data of patients with MAP and MSAP versus SAP. Table 3 and Figure 2 present the area under the curve (AUC), the ideal cut-off value, SE, SP, PPV, NPV, +LR, and −LR of each prognostic variable for SAP.

**Table 1:** Demographics, mortality, and in-hospital outcomes in patients with AP.

	MAP (n = 124)	MSAP (n = 67)	SAP (n = 25)
Male (%)	61 (49%)	41 (61%)	18 (72%)
Mean age (yr)	56.8	53.4	61.9
Aetiology % (nr of patients/total nr)			
Biliary	57% (71/124)	50% (34/67)	60% (15/25)
Alcohol	18% (23/124)	23% (16/67)	0% (0/25)
Hypertriglyceridemia	4% (5/124)	11% (7/67)	16% (4/25)
Other	20% (25/124)	13% (10/67)	24% (6/25)
Mortality (%)	0	0	19 (76%)
Mean hospitalization length (d)	5.5	8.3	18.5
ICU admission % (nr of patients/total nr)	0	0	84% (21/25)
OIT % (nr of patients/total nr)	0	0	84% (21/25)
HHD % (nr of patients/total nr)	0	0	4% (1/25)
Emergency surgery % (nr of patients/total nr)	0	1% (1/67)	48% (12/25)
Other interventions			
Percutaneous aspiration % (nr of patients/total nr)	0	1% (1/67)	4% (1/25)
DFPP for HTG% (nr of patients/total nr)	0	1% (1/67)	4% (1/25)

DFPP = double filtration plasmapheresis, HHD = hemodialysis, HTG = hypertriglyceridemia, ICU = intensive care unit, MAP = mild acute pancreatitis, MSAP = moderately severe acute pancreatitis, OIT = orotracheal intubation, SAP = severe acute pancreatitis.

**Table 2:** Laboratory data of patients with MAP+MSAP vs SAP.

	MAP+MSAP mean±SD	SAP mean±SD	*P*
BISAP	2±1	3 ± 1	<.0001
Creatinine	0.9 ± 0.7	2 ± 1.5	<.0001
Creatinine48h	1 ± 1	2.5 ± 1.9	<.0001
Ht	42 ± 5	40 ± 8	.08
Ht48h	38 ± 5	35 ± 6	.006
CRP48h	103 ± 101	235 ± 135	<.0001
NLR	8 ± 10	13 ± 8.5	.01
NLR48h	6 ± 4	11 ± 4	<.0001
RDW	13 ± 1	13 ± 2	1
BUN	41 ± 29	73 ± 50	<.0001
BUN48h	39 ± 36	88 ± 65	<.0001
PLR	166 ± 92	224 ± 178	.01
PLR48h	152 ± 126	193 ± 88	.1
Blood glucose level	152 ± 81	195 ± 88	.005
Lipase level	11013 ± 22756	11786 ± 14052	.8

BUN = blood urea nitrogen, CRP = C-reactive protein, Ht = hematocrit, MAP = mild acute pancreatitis, MSAP = moderately severe acute pancreatitis, NLR = neutrophil-to-lymphocyte ratio, PLR = platelet-to-lymphocyte ratio, RDW = red cell distribution width, SAP = severe acute pancreatitis, SD = standard deviation.

**Table 3:** Prognostic value of BISAP score and serum markers for predicting SAP.

	Cut off value	SE	SP	AUC	*P*	PPV	NPV	LR+	LR−
BISAP	>2	61%	88%	0.77	<.0001	32%	94%	5	0.4
Creatinine	>1.1	61%	84%	0.74	<.0001	33%	94%	3.8	0.4
Creatinine48h	>1.4	60%	91%	0.79	<.0001	47%	94%	6.6	0.4
Ht	<37.1	38%	81%	0.54	.54	21%	90%	2	0.7
Ht48h	<35.2	59%	77%	0.64	.04	25%	93%	2.5	0.5
CRP48h	>217	71%	88%	0.8	<.0001	44%	91%	5.9	0.3
NLR	>9.6	65%	70%	0.68	.002	22%	83%	2.1	0.5
NLR48h	>6.15	100%	63%	0.83	<.0001	26%	100%	2.7	0
RDW	>13.6	45%	75%	0.58	.2	19%	85%	1.8	0.7
BUN	>56	53%	89%	0.73	.0002	39%	88%	4.8	0.5
BUN48h	>47	78%	80%	0.79	<.0001	34%	96%	3.9	0.2
PLR	>157	69%	53%	0.59	.15	16%	92%	1.4	0.5
PLR48h	>92.6	100%	25%	0.64	.02	15%	92%	1.3	0
Blood glucose level	>176	53%	82%	0.67	.003	28%	92%	2.9	0.5

AUC = area under the curve, BUN = blood urea nitrogen, CRP = C-reactive protein, Ht = hematocrit, LR+ = positive likelihood, LR− = negative likelihood, NLR = neutrophil-to-lymphocyte ratio, NPV = negative predictive value, PLR = platelet-to-lymphocyte ratio, PPV = positive predictive value, RDW = red cell distribution width, SE = sensibility, SP = specificity.

The AUC for the BISAP score was fair. Among the serum markers, NLR48 h and CRP48 h had the highest AUCs. NLR48 h had 100% SE and 63% SP, and CRP48 h had 71% SE and 88% SP. The creatinine level at 48 hours had the highest PPV (47%). All parameters had a very good NPV; NPV was 100% for NLR48 h. The creatinine level at 48 hours had the highest +LR (6.6), followed by CRP48 h. The −LR value was 0 for NLR48 h and PLR48 h and 0.2 for BUN at 48 hours.

CRP48 h (*P* = .004) and NLR48 h (*P* = .003) were independently associated with SAP in the multiple regression analysis (Table 4). Thus, we assessed CRP48 h and NLR48 h in tandem, and the AUC was 0.89, the *P* value was < .0001, the SE was 68%, and the SP was 92%.

**Table 4:** Least-squares multiple regression.

Independent variables	Coefficient	Std. error	R partial	*t*	*P*
(Constant)	−0.1476				
CRP48h	0.0009131	0.0002466	0.3133	3.703	.0004
NLR48h	0.02535	0.006859	0.3127	3.696	.0003

CRP = C-reactive protein, NLR = neutrophil-to-lymphocyte ratio.

Regarding the prognostic accuracy of serum marker changes over 48 hours (Table 5), a creatinine level decrease of less than 0.3 mg/dL after 48 hours may indicate SAP development. Further, if BUN does not drop with 7 mg/dL after 48 hours, this may also indicate SAP development.

**Table 5:** Dynamics of serum markers for the prediction of SAP.

	Cut off	SE	SP	AUC	*P*
Cr-Cr48h	<−0.3	42%	94%	0.65	.03
BUN-BUN48h	<−7	53%	91%	0.67	.02
Ht-Ht48h	>41	62%	59%	0.5	.7
NLR-NLR48h	<−0.3	52%	70%	0.5	.5

AUC = area under the curve, BUN = blood urea nitrogen, Cr = creatinine, Ht = hematocrit, NLR = neutrophil-to-lymphocyte ratio, SE = sensibility, SP = specificity.

MSAP and SAP patients were analyzed as 1 group to determine if the relevant variables could predict the overall complication rate (Table 6). In this case, the parameters were less reliable; the highest AUCs were 0.77 for CRP (PPV: 65%, NPV: 76%) and 0.76 for NLR48H (PPV: 67%, NPV: 78%).

**Table 6:** Prognostic value of BISAP score and serum markers for predicting complications.

	Cut-off value	SE	SP	AUC	*P*	PPV	NPV
BISAP	>2	40%	91%	0.67	<.0001	76%	67%
Creatinine	>0.7	73%	52%	0.64	.0001	52%	72%
Creatinine48h	>0.9	54%	82%	0.68	.0002	68%	70%
Ht	>39.2	71%	36%	0.5	.8	44%	62%
Ht48h	<35.2	40%	81%	0.6	.02	60%	64%
CRP48h	>93	68%	74%	0.77	<.0001	65%	76%
NLR	>5	78%	38%	0.59	.01	47%	70%
NLR48h	>6.1	72%	75%	0.76	<.0001	67%	78%
RDW	>13.8	27%	80%	0.5	.6	49%	60%
BUN	>54	28%	90%	0.5	.005	67%	63%
BUN48h	>48	45%	91%	0.6	.001	78%	69%
PLR	<93	26%	81%	0.5	.8	49%	59%
PLR48h	>189	29%	90%	0.5	.04	67%	63%
Blood glucose level	>166	37%	84%	0.6	.009	62%	64%

AUC = area under the curve, BUN = blood urea nitrogen, CRP = C-reactive protein, Ht = hematocrit, NLR = neutrophil-to-lymphocyte ratio, NPV = negative predictive value, PLR = platelet-to-lymphocyte ratio, PPV = positive predictive value, RDW = red cell distribution width, SE = sensibility, SP = specificity.

Overall, 199 patients survived, and 19 did not. Multiple regression analysis indicated that only CRP (*P* = .003) and Ht at 48 hours (*P* = .01) were independently associated with mortality.

## 4 Discussion

In this study, the proportion of patients with complicated AP was higher than that reported in the literature. The overall complication rate (MSAP and SAP) was 42%, and 11% of cases had SAP, while others reported complications in 20% of patients,^[18]^ and the proportion of SAP was 7.7% lower than in our study.^[3]^ The discrepancies between the rates were likely because our hospital is a tertiary care facility, and some patients were directly referred from the surrounding area.

Mortality among patients with SAP was higher in our study (76%) than in the literature.^[19]^ This result emphasizes the necessity to identify at-risk patients for appropriate monitoring and improved outcomes, which could be facilitated by simplifying predicting modalities. In our study, patients were rarely admitted to the intensive care unit owing to logistical issues and competition with other wards also requiring beds, such as neurosurgery, which may explain the high mortality rate.

There is considerable interest in developing rapid biomarkers for reliable prognosis predictions for AP, which can guide disease management and improve outcomes. The biomarker levels at admission were the most critical. However, at this point, they had only an acceptable predictive value. The BISAP score had the best AUC (0.77), followed by creatinine (0.74) and BUN (0.73). Further, the accuracy of these biomarkers increased after 48 hours, mirroring the response to fluid resuscitation and the inflammatory response severity.

The predictive value of the NLR has been reported for colorectal, lung, and pancreatic cancers. Recently, many studies have reported that NLR is not only a cancer-specific prognostic factor but also a prognostic factor for systemic inflammatory diseases (e.g., bacteremia), surgery outcomes, and acute kidney injury.^[3]^ In our study, NLR48 h had the highest accuracy for predicting SAP and mortality, outperforming the other laboratory parameters and the BISAP score. Jeon and Park^[5]^ also reported that NLR48 h had a higher AUC for predicting severity than baseline values (AUC: 0.62 vs 0.59).

Our NLR cut-off values at admission for predicting SAP (>9.6) were higher than those reported by Azab et al^[20]^ (>4.7) and Jeon and Park^[5]^ (>6.14). However, Kaplan et al^[10]^ reported a cut-off value of > 13.64 for predicting SAP. Further, Kokulu et al^[2]^ reported a baseline cut-off value of > 7.13 and a 48-hour value of > 6.2 with an AUC of 0.93. In our study, the ideal cut-off value at 48 hours was 6.16 with an AUC of 0.84. The differences between studies may be owing to the heterogeneous study population and differences in classifying AP severity. Our results suggested that NLR48 h was independently associated with SAP, as was the CRP level. Li et al^[19]^ also demonstrated that the CRP level was an independent predictor of SAP.

NLR and CRP have the same disadvantage; they peak after the initial critical 24 to 48-hour period. However, when we combined both parameters as a prediction tool, the ability to predict SAP was higher than any other single parameter or scoring system analyzed (AUC: 0.89, SE: 68%, and SP: 92%).

The creatinine level is also important, and acute kidney injury is one of the most frequent complications in patients with AP. Our results indicated that the creatine level was an acceptable predictor at admission and after 48 hours. Our attempts to validate the role of hemoconcentration failed to confirm the accuracy of this test as a prognostic marker in AP, likely owing to the heterogeneity of our patient populations. Wu et al^[21]^ have also demonstrated a weak correlation between the hematocrit level and the severity of AP. We also could not find a prognostic relationship between PLR and AP severity, supporting Ilhan et al, who also reported no correlation. Although glucose level is a component of the Ranson score, we did not identify a correlation between the glucose level and AP severity, nor did we find a correlation with the RDW level.

In clinical settings, +LR and −LR are useful tools. The creatine level at 48 hours, CRP48 h, and the BISAP score had the highest +LR, suggesting a moderate increase in the chance of developing SAP. NLR48 h and PLR48 h had a −LR score of 0.

BUN could be useful as a surrogate marker of intravascular volume status to evaluate the effectiveness of initial resuscitation efforts. Wu et al^[22]^ determined that serial BUN measurements were more accurate in predicting SAP. An increase in the BUN level of 5 mg/dL or more at 48 hours was one of 11 criteria originally established as part of the Ranson score. Based on this, we aimed to improve the performance of the BUN level at baseline (AUC: 0.73) and 48 hours (AUC: 0.77), so we analyzed the trend. We found that if BUN does not decrease to 7 mg/dL after 48 hours, it may predict SAP development (AUC: 0.67). The same analyses for NLR and Ht yielded no significant results.

In this study, NLR48 h (AUC: 0.77) and CRP (AUC: 0.76) were predictors for overall complications. However, these markers were less reliable, perhaps because MSAP and MAP do not differ considerably, and the inflammatory response is milder than in SAP. CRP was an independent predictor of mortality, in line with the results of Li et al. However, our analyses also found that Ht at 48 hours was associated with higher mortality.

This study had several limitations. The retrospective, nonrandomized, and single-center design may have resulted in selection bias. Further, patients with incomplete clinical data were not enrolled, which could have led to incomplete analyses.

In conclusion, NLR48 h and CRP48 h were independently associated with SAP, and BUN had a good predictive performance. Combining NLR48 h and CRP48 h was the best method for predicting SAP. However, the CRP level was a good predictive marker for mortality. The above-mentioned markers evaluated at admission were not superior to the BISAP score in predicting SAP. We suggest using these simple, affordable laboratory tests as prognostic biomarkers in patients admitted to the emergency department with AP to identify severe forms.

## Author contributions

**Conceptualization:** Greta Maria Dancu, Alina Popescu, Roxana Sirli, Mirela Danila, Felix Bende, Cristi Tarta, Ioan Sporea.

**Data curation:** Greta Maria Dancu, Alina Popescu, Ioan Sporea.

**Formal analysis:** Greta Maria Dancu, Alina Popescu, Cristi Tarta, Ioan Sporea.

**Investigation:** Greta Maria Dancu, Alina Popescu, Mirela Danila, Cristi Tarta, Ioan Sporea.

**Methodology:** Greta Maria Dancu, Alina Popescu, Roxana Sirli, Mirela Danila, Felix Bende, Ioan Sporea.

**Project administration:** Ioan Sporea.

**Resources:** Greta Maria Dancu, Felix Bende, Ioan Sporea.

**Supervision:** Greta Maria Dancu, Alina Popescu, Ioan Sporea.

**Validation:** Greta Maria Dancu, Alina Popescu, Roxana Sirli, Mirela Danila, Felix Bende, Cristi Tarta, Ioan Sporea.

**Visualization:** Greta Maria Dancu, Alina Popescu, Roxana Sirli, Cristi Tarta, Ioan Sporea.

**Writing – original draft:** Greta Maria Dancu, Alina Popescu, Roxana Sirli, Mirela Danila, Felix Bende, Cristi Tarta, Ioan Sporea.

**Writing – review & editing:** Greta Maria Dancu, Alina Popescu, Roxana Sirli, Mirela Danila, Felix Bende, Cristi Tarta, Ioan Sporea.
